# Spatial, Temporal, and Habitat-Related Variation in Abundance of Pelagic Fishes in the Gulf of Mexico: Potential Implications of the Deepwater Horizon Oil Spill

**DOI:** 10.1371/journal.pone.0076080

**Published:** 2013-10-10

**Authors:** Jay R. Rooker, Larissa L. Kitchens, Michael A. Dance, R. J. David Wells, Brett Falterman, Maëlle Cornic

**Affiliations:** 1 Department of Marine Biology, Texas A&M University, Galveston, Texas, United States of America; 2 Department of Wildlife and Fisheries Sciences, Texas A&M University, College Station, Texas, United States of America; 3 McDaniel Charitable Foundation, Texas City, Texas, United States of America; Aristotle University of Thessaloniki, Greece

## Abstract

Time-series data collected over a four-year period were used to characterize patterns of abundance for pelagic fishes in the northern Gulf of Mexico (GoM) before (2007–2009) and after (2010) the Deepwater Horizon oil spill. Four numerically dominant pelagic species (blackfin tuna, blue marlin, dolphinfish, and sailfish) were included in our assessment, and larval density of each species was lower in 2010 than any of the three years prior to the oil spill, although larval abundance in 2010 was often statistically similar to other years surveyed. To assess potential overlap between suitable habitat of pelagic fish larvae and surface oil, generalized additive models (GAMs) were developed to evaluate the influence of ocean conditions on the abundance of larvae from 2007–2009. Explanatory variables from GAMs were then linked to environmental data from 2010 to predict the probability of occurrence for each species. The spatial extent of surface oil overlapped with early life habitat of each species, possibly indicating that the availability of high quality habitat was affected by the DH oil spill. Shifts in the distribution of spawning adults is another factor known to influence the abundance of larvae, and the spatial occurrence of a model pelagic predator (blue marlin) was characterized over the same four-year period using electronic tags. The spatial extent of oil coincided with areas used by adult blue marlin from 2007–2009, and the occurrence of blue marlin in areas impacted by the DH oil spill was lower in 2010 relative to pre-spill years.

## Introduction

Pelagic fishes that frequent open oceans are valuable economically and influence the structure and resilience of offshore ecosystems [1]–[2]. Declines in the abundances of fishes in nearshore and offshore ecosystems have been shown to limit top-down regulation of marine food webs, often leading to reductions in ecosystems services [3]–[5]. Anthropogenic drivers of ecosystem change in the open ocean have been linked to a variety of factors that alter the relative abundance of fishes, including overfishing [6], climate change [7], and oil pollution [8]. Of these, the ecological consequences of oil pollution from massive spills are largely undetermined.

In April 2010, the Deepwater Horizon [DH) oil spill resulted in the release of approximately 4.4×10^6^ (±20%) barrels of oil into the northern Gulf of Mexico (GoM) [9). Oil trajectory models predicted that surface or near surface oil covered a large region of the continental shelf and slope [10]. In the past few years, several studies have attempted to assess the ecological impacts of the DH oil spill on coastal and estuarine ecosystems in the GoM, including research on higher order consumers such as fishes [11]. While the need to understand the impacts to inshore areas is obvious, it is also important to recognize that oceanic waters south of the DH oil spill experienced significant exposure to both oil and dispersants [12]. Moreover, slope waters south of the DH oil spill represent an important hotspot of productivity for oceanic fishes, serving as both nursery and spawning habitat of several pelagic fishes [13]–[14]. Given that many oceanic fishes reside in epipelagic regions of the water column and spawn in the late spring and summer when surface slicks from the DH oil spill were widespread in 2010, there is a clear need to investigate population-level responses of pelagic fishes to this event, particularly during the vulnerable early life stage.

The aim of the present study was to assess possible impacts of the DH oil spill on four pelagic fishes that commonly inhabit outer shelf and slope waters of the northern GoM: blackfin tuna (*Thunnus atlanticus*), blue marlin (*Makaira nigricans*), dolphinfish (*Coryphaena hippurus*), and sailfish (*Istiophorus platypterus).* We first explored temporal variability during early life by contrasting larval abundances of each species before (2007–2009) and after (2010) the DH event. Next, generalized additive models (GAMs) were developed from 2007–2009 data to assess habitat associations, and then to predict the probability of occurrence the following year (2010) in relation to surface oil (i.e., overlap). Finally, patterns of occurrence for a model pelagic predator (blue marlin) over the same four-year period were examined using pop-up archival transmitting (PAT) tags, and light-based geo-locations from tags were used to compare the distribution of adults before and after the DH oil spill. Our working hypothesis was that the distribution and abundance of pelagic fishes in our study area, both early life and adult stages, may have changed in 2010 due to changes in habitat condition or other activities related to the DH oil spill. We fully recognize that natural environmental variability is known to influence the distribution and abundance of pelagic fishes in the northern GoM [13], limiting our ability to directly link any temporal changes in distribution or abundance directly to the oil spill.

## Results

Of the four taxa examined, blackfin tuna were numerically dominant and this species accounted for 87% of the total catch of selected taxa (Table 1). Dolphinfish and sailfish larvae were also relatively well represented, although the abundance of these taxa was an order of magnitude lower than blackfin tuna. Blue marlin larvae were present at the lowest density. Percent occurrence was highest for blackfin tuna (81%) and dolphinfish (57%), with larvae of both species being widely distributed throughout our sampling corridor (Fig. S1). Percent occurrence was considerably lower for billfish larvae: sailfish (28%), blue marlin (18%).

**Table 1:** Summary data on the density [larvae⋅ 1000 m^−3^ (±1 standard deviation)] and percent occurrence of blackfin tuna (*Thunnus atlanticus*), blue marlin (*Makaira nigricans),*dolphinfish (*Coryphaena hippurus*), and sailfish (*Istiophorus platypterus*).

			Blackfin tuna			Dolphinfish		
Year	Month	Stations	N	Density (SD)	% Freq	N	Density (SD)	% Freq
2007	June	59	1208	8.43 (12.54)	90	235	1.72 (3.44)	71
2007	July	55	3048	24.75 (34.88)	96	61	0.48 (0.67)	51
2008	June	72	1135	6.90 (10.16)	72	110	0.64.04)	50
2008	July	76	854	4.80 (10.19)	61	87	0.51 (0.67)	55
2009	June	49	540	5.05 (8.21)	84	132	1.26 (1.61)	82
2009	July	77	4430	30.63 (61.31)	91	110	0.73 (1.97)	39
2010	June	48	657	4.74 (9.76)	67	58	0.42 (0.70)	48
2010	July	48	1012	7.13 (14.92)	83	61	0.45 (0.50)	60
			**Blue marlin**			**Sailfish**		
**Year**	**Month**	**Stations**	**N**	**Density (SD)**	**% Freq**	**N**	**Density (SD)**	**% Freq**
2007	June	59	62	0.37 (0.93)	25	113	0.75 (3.52)	22
2007	July	55	97	0.77 (1.52)	38	65	0.57 (1.34)	31
2008	June	72	10	0.05 (0.25)	7	196	1.03 (2.95)	33
2008	July	76	61	0.34 (0.80)	26	83	0.52 (2.31)	24
2009	June	49	11	0.11 (0.57)	6	143	1.40 (3.08)	47
2009	July	77	78	0.46 (1.84)	10	80	0.55 (1.27)	30
2010	June	48	4	0.03 (0.11)	6	18	0.13 (0.31)	21
2010	July	48	19	0.14 (0.31)	25	18	0.13 (0.42)	19

Larvae were collected during ichthyoplankton surveys conducted in the northern Gulf of Mexico from 2007 to 2010. Numbers of stations sampled each survey are also provided.

**Figure 1:**
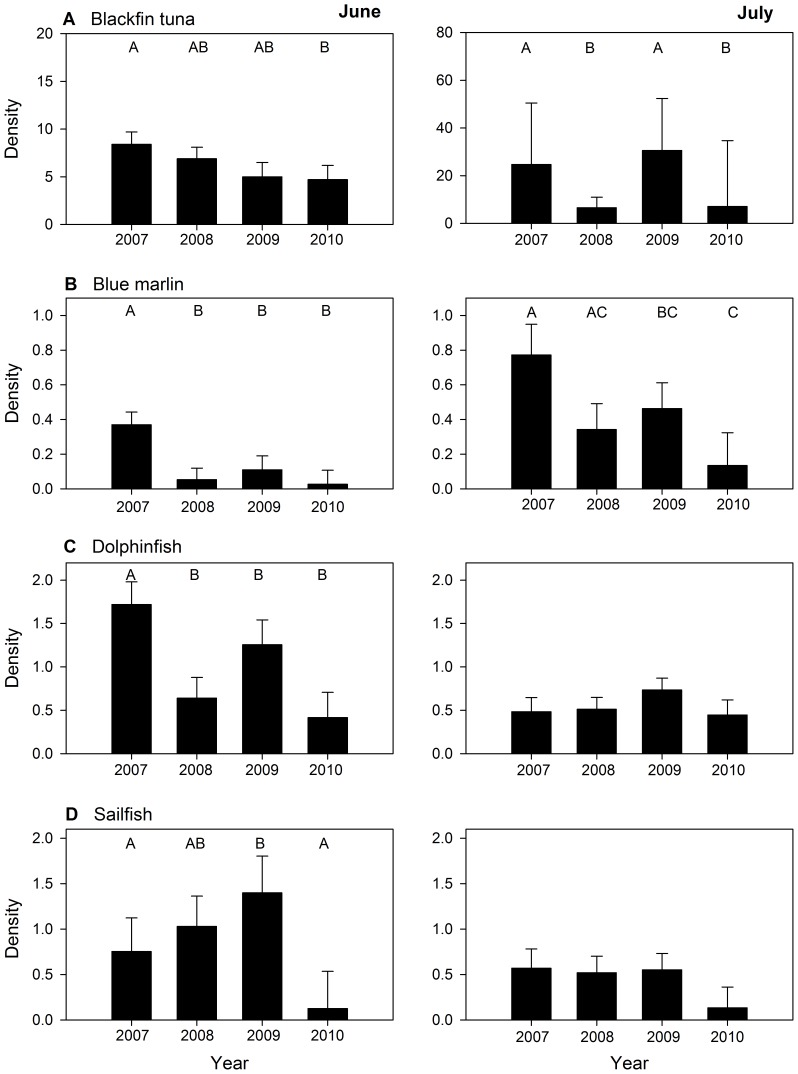
Mean density [larvae ⋅ 1000 m^−3^ (±1 standard error)] of pelagic fish larvae collected from 2007 to 2010 for A) blackfin tuna (*Thunnus atlanticus*), B) blue marlin (*Makaira nigricans),* C) dolphinfish (*Coryphaena hippurus*), and D) sailfish (*Istiophorus platypterus*). Left and right panels represent results for June and July surveys, respectively. Upper case letters on plots represent significant differences based on multiple comparisons with Wilcoxon non-parametric test (p<0.05).

Due to the presence of short-term temporal differences in both density (i.e., month effect) and the oceanographic factors that influence the abundance of larvae, inter-annual variability was investigated with separate models for June and July. In June surveys, a significant year effect was observed for all four species: blackfin tuna (p = 0.030), blue marlin (p = 0.001), dolphinfish (p = <0.001), and sailfish (p = 0.006). Despite the fact that the mean density of each species was lower in June 2010 than any of the other June surveys (Table 1), this year was often statistically similar to other pre-spill year(s) (Fig. 1). In July surveys, a significant year effect was also observed for blackfin tuna (p<0.001) and blue marlin (p = 0.003). Again, the lowest observed density present among July surveys occurred in 2010 for three species (blue marlin, dolphinfish, and sailfish; Table 1); however, non-parametric comparisons indicated that 2010 was statistically similar to other years examined (Fig. 1).

GAMs based on presence/absence data developed for each species indicated that the influence of 7 oceanographic factors (chlorophyll *a* concentration, depth, distance to feature, feature classification, salinity, sea surface temperature, sea surface height anomaly) on the occurrence of larvae varied across species and within species when contrasting June and July models (Table 2, Fig. S2, S3). Final GAMs for blackfin tuna (June AIC = 139.3, July AIC = 182.8) retained four explanatory variables for both June and July, with sea surface temperature and salinity common to both models. Percent deviance explained for blackfin tuna GAMs was 30.4% (June) and 15.7% (July). Final GAMs for blue marlin (June AIC = 66.3, July AIC = 141.7) also included four explanatory variables in both models. Sea surface temperature, chlorophyll *a* concentration, and depth were influential parameters common to both June and July models, and percent deviance explained for blue marlin GAMs was 62.5% (June) and 44.3% (July). Final GAMs for dolphinfish (June AIC = 213.0, July AIC = 267.1) retained three explanatory variables in the June model and four in the July model, with depth being the only variable common between the two models. Percent deviance explained for dolphinfish GAMs was 13.2% (June) and 13.5% (July). Final GAMs for sailfish (June AIC = 199.5, July AIC = 221.9) included three common explanatory variables in June and July models (sea surface height anomaly, salinity, distance to mesoscale feature), and percent deviance explained was 20.3% (June) and 15.3% (July).

**Table 2:** Explanatory variables retained in final presence/absence generalized additive models (GAMs) for blackfin tuna (*Thunnus atlanticus*), blue marlin (*Makaira nigricans),* dolphinfish (*Coryphaena hippurus*), and sailfish (*Istiophorus platypterus*).

June Models	Blackfin tuna		Blue marlin		Dolphinfish		Sailfish	
	AIC = 139.3, DE = 30.4%		AIC = 66.3, DE = 62.5%		AIC = 213.0, DE = 13.2%		AIC = 199.5, DE = 20.3%	
Variable	GAM	Δ AIC	GAM	Δ AIC	GAM	Δ AIC	GAM	Δ AIC
Sea surface temperature	⧫	9.8	⧫	14.5				
Sea surface height anomaly	⧫	19.2			⧫	10	⧫	23.6
Chlorophyll *a*			⧫	20.6			⧫	5.3
Salinity	⧫	5.6	⧫	4.9	⧫	6.3	⧫	3.6
Depth			⧫	11	⧫	4.6		
Distance to feature	⧫	15.8					⧫	2.3
Feature classification								
**July Models**	**Blackfin tuna**		**Blue marlin**		**Dolphinfish**		**Sailfish**	
	**AIC = 182.8, DE = 15.7%**		**AIC = 141.7, DE = 44.3%**		**AIC = 267.1, DE = 13.5%**		**AIC = 221.9, DE = 15.3%**	
**Variable**	**GAM**	**Δ AIC**	**GAM**	**Δ AIC**	**GAM**	**Δ AIC**	**GAM**	**Δ AIC**
Sea surface temperature	⧫	17.4	⧫	2.3				
Sea surface height anomaly			⧫	3			⧫	11.1
Chlorophyll *a*			⧫	18.6	⧫	12.8		
Salinity	⧫	1.8					⧫	23.1
Depth	⧫	2.7	⧫	7.7	⧫	2.3		
Distance to feature					⧫	2.4	⧫	7.2
Feature classification	⧫	1.9			⧫	8.9		

Akaike’s Information Criterion (AIC) and percent deviance explained (DE) are given for each final model. ΔAIC values are based on the difference if the variable was excluded from the final model.

**Figure 2:**
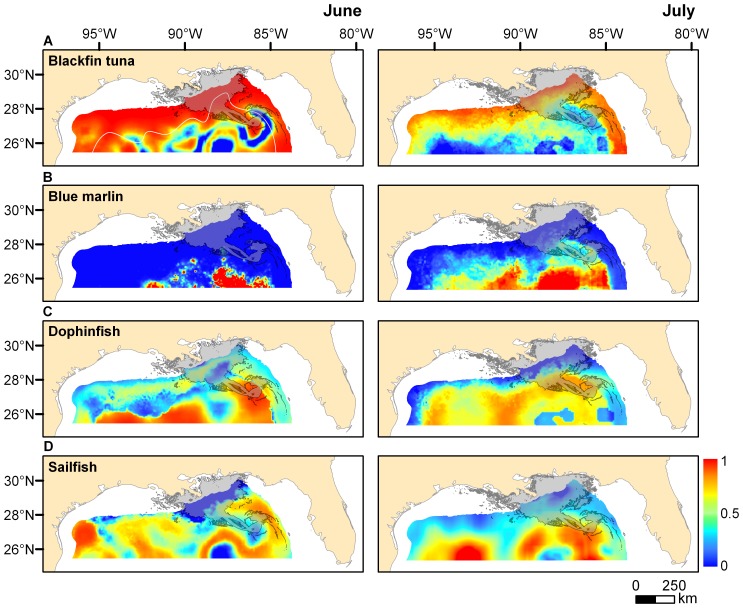
Predicted probability of occurrence for larvae of each species in offshore waters (>100 m) of the Gulf of Mexico. Spatial distributions are based on using explanatory variables from GAMs (based on 2007–2009 data) to predict the probability of occurrence in 2010 for A) blackfin tuna (*Thunnus atlanticus*), B) blue marlin (*Makaira nigricans),* C) dolphinfish (*Coryphaena hippurus*), and D) sailfish (*Istiophorus platypterus*). Left and right panels represent predictions for June and July, respectively. Gray shading on plots represents the estimated cumulative extent of surface oil on both June 15^th^ (June plots) and July 30^th^ (July plots), 2010 derived from daily oil spill coverage accessed from www.nesdis.noaa.gov and combined within ArcGIS. Continental shelf (<100 m) represented in areas without color and 2000 m depth contour also shown (white line) on plot A.

The spatial distribution of suitable habitat in 2010, predicted with GAMs developed from 2007–2009 data, indicated that the geographic location and areal coverage of high quality habitat (defined here as probability of occurrence ≥0.50) varied markedly among species (Fig. 2). The spatial extent of predicted high quality habitat of blackfin tuna and dolphinfish was broader than either billfish species, encompassing 331,224–402,791 km^2^ and 262,869–334,879 km^2^, respectively (Table 3). High quality habitat of blackfin tuna occurred across large areas of outer shelf to upper slope (100–1000 m) and lower slope (1000–2000 m), while high quality habitat of dolphinfish was typically in the deeper abyssal region (>2000 m) or south of 28°N. High quality habitat of sailfish was also generally located in slope and abyssal regions of the northern GoM, covering between 189,172–276,384 km^2^ in the area examined. The distribution of high quality habitat for blue marlin was more limited than the other species examined and present primarily in abyssal regions.

**Table 3:** Estimated areal coverage (km^2^) of high quality (HQ) early life habitat available for blackfin tuna (*Thunnus atlanticus*), blue marlin (*Makaira nigricans),* dolphinfish (*Coryphaena hippurus*), and sailfish (*Istiophorus platypterus*) in both June and July, 2010.

	June			July		
Species	HQ Habitat(km^2^)	HQ HabitatExposed (km^2^)	Percent Overlap	HQ Habitat(km^2^)	HQ HabitatExposed (km^2^)	Percent Overlap
Blackfin tuna	402,791	61,529	15.3%	331,224	64,146	19.4%
Blue marlin	9,464	0	0.0%	92,444	200	0.2%
Dolphinfish	334,879	46,561	13.9%	262,869	29,868	11.4%
Sailfish	276,384	12,233	4.5%	189,172	13,549	7.2%

Areal coverage of surface oil within HQ habitat also provided along with estimates of percent overlap between HQ habitat and surface oil. HQ habitat defined as probability of occurrence ≥0.50.

The spatial extent of surface oil from the DH event was viewed in relation to the total available area of high quality habitat predicted with GAMs to determine whether suitable habitat of each species was exposed to oil contamination. In June 2010, the estimated overlap between high quality habitat and surface oil was relatively low for blue marlin (0%) and sailfish (4.5%). Conversely, percent overlap for blackfin tuna and dolphinfish was considerably higher, with 15.3% and 13.9% of the predicted high quality habitat exposed to surface oil in the northern GoM. Species-specific patterns for July 2010 were similar, and again the percentage of high quality habitat exposed to surface oil was considerably lower for blue marlin (0.2%) and sailfish (7.2%) when compared to blackfin tuna (19.4%) or dolphinfish (11.4%). Given the predicted overlap observed for blackfin tuna, it is not surprising that the areal coverage of high quality habitat exposed to surface oil was relatively large, exceeding 60,000 km^2^ in both June and July.

**Figure 3:**
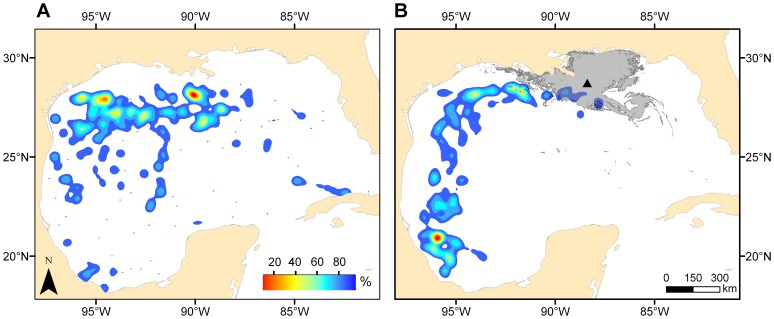
Habitat utilization of adult blue marlin (*Makaira nigricans*) generated from electronic tag deployments A) before (2007–2009) and B) after (2010) the DH oil spill. Probability density (cell size = 0.01°) was calculated from daily mean position estimates during the months of May to July (spawning season). For clarity, only 90% of the utilization distribution was projected. ▴ denotes location of Deepwater Horizon platform. Gray shading on plots represents cumulative oil coverage through July 30^th^ 2010, derived from daily oil spill coverage accessed from www.nesdis.noaa.gov and combined within ArcGIS.

Geo-location estimates from electronic tag deployments on adult blue marlin during the May-July spawning period were used to determine their spatial distribution before (2007–2009) and after (2010) the DH oil spill (Fig. 3). From 2007–2009, core use areas (based on 50% kernel utilization distribution [KUD]) of adult blue marlin were present throughout the northwestern and northcentral GoM, while 50% KUD in 2010 was primarily limited to the western GoM. Estimates of area from 50% KUD before (2007–2009) the DH oil spill were coupled with the location of surface oil to determine whether suitable spawning habitat may have been exposed to surface oil. Our overlap analysis indicated that 1,034 km^2^ (9.3%) of the core use area in 2007–2009 was exposed to surface oil in 2010. Based on daily mean positions, we also observed that use of a region subsequently closed to fishing in 2010 by the National Oceanic and Atmospheric Administration (based on June 21, 2010 closure due to the DH oil spill; Fig. S4) was twofold higher in 2007–2009 (16.4%) compared to 2010 (8.2%), possibly indicating lower occurrence of adult blue marlin in areas proximal to the DH oil spill in 2010.

## Discussion

Both the abundance and percent occurrence of fish larvae for selected pelagic fishes declined in 2010 relative to the three years prior to the DH oil spill, suggesting that changes in environmental conditions, possibly linked to the presence of oil and dispersants, may have contributed to observed inter-annual variability. Previous studies have demonstrated a clear relationship between these contaminants and the demographics of a wide range of marine organisms including invertebrates, fishes, birds, and mammals [15]–[17]. Early life stages of invertebrates and fishes with a pelagic larval phase are particularly vulnerable to oil spills because toxic compounds accumulate in near surface waters (epipelagic zone) where larvae reside. In addition, the same physical processes that transport larvae also transport oil and dispersants, which may increase the probability of exposure [11]. For fish, exposure to toxic compounds in oil such as polycyclic aromatic hydrocarbons (PAHs) result in a variety of different adverse biological effects including genotoxicity, oxidative stress, and growth retardation [18]–[20]. The disruption of developmental processes due to early life exposure to PAHs and dispersants is also well documented [21]–[22], and altered functions during the embryonic or larval period are often lethal [16], leading to weakened cohort strength and potential recruitment failure. The most conspicuous declines in 2010 were observed for billfish larvae (sailfish, blue marlin), which reside primarily in the epipelagic zone. Given that the larvae of these species are typically restricted to surface waters relative to other taxa surveyed (i.e., tunas), it is possible that their increased exposure to toxic compounds may have affected early life survival.

Although results from this study suggest a possible connection between reduced abundance of pelagic fish larvae in 2010 and the DH oil spill, inter-annual variability is relatively common for cohorts of pelagic larvae in the GoM [13], [23]. The apparent decline in billfish, dolphinfish, and tuna larvae therefore may be due in part to shifts in biological or oceanographic conditions unrelated to the DH oil spill. Several studies have reported significant changes in larval biomass or abundance from year to year, and recruitment failures have been linked to a variety of natural conditions including mismatches between zooplankton (prey) and fish larvae [24]–[25], unfavorable meteorological or hydrodynamic processes [26]–[27], and shifts in physicochemical conditions [28]. Because the pre-spill data used here were limited in duration (2007–2009), our baseline may not have adequately represented the entire range of biological and oceanographic conditions commonly experienced by cohorts of pelagic fish larvae in the GoM. It is also important to note that abundances in 2010 were often found to be statistically similar to other year(s) in our baseline. Moreover, temporal variability in the abundance of certain species were in phase (i.e., low abundance blackfin tuna and blue marlin in 2008 and 2010), suggesting that observed declines were at least within the range of expected natural variation for these taxa, and thus may not be related to the DH oil spill.

Variability in mesoscale circulation has been shown to influence the spatial distribution of larvae in the GoM [13], which in turn can affect the abundance of pelagic larvae collected from surveys that cover the same relative areas year after year [29]. In particular, larvae of several pelagic taxa occur at higher densities near the margin of the Loop Current (LC) in the GoM, and the spatial dynamics of this large anticyclonic feature varies seasonally and across years. The northern and western penetration of the LC has been shown to influence the abundance of larvae for several of the taxa examined here [30], and therefore temporal differences in larval abundance may be linked to inter-annual variability in the penetration and shape of the LC. During June and July 2010, the northward penetration of the LC and associated features (anticyclonic eddies shed from the LC) was slightly less than observed during the same periods in 2007–2009, with little evidence of anticyclonic conditions north of 27°N in the GoM (Fig. S1). Consequently, fewer stations in 2010 were located on or near the margins of anticyclonic features relative to other years, which may have contributed to the reduced densities observed in 2010.

Determining the primary cause of annual fluctuations in the abundance of pelagic fish larvae is challenging, and establishing a direct link between the abundance of larvae and the DH oil spill is not possible given the data available. However, our estimates of overlap between surface oil and high quality habitat of billfish, dolphinfish, and tuna larvae, support the premise that highly suitable nursery areas (or spawning areas) of these taxa were impacted by the DH oil spill. Oil contamination occurred across several hundred thousand square kilometers of suitable habitat of pelagic fish larvae, accounting for approximately 10–20% of the available high quality habitat for blackfin tuna and dolphinfish in the northern GoM. Estimates of percent overlap were lower for billfish larvae (blue marlin, sailfish), and suitable habitat for these taxa appeared to be more limited in coverage and farther offshore (i.e., slope to abyssal regions), possibly out of reach of significant oil contamination from the DH oil spill. While the presence of surface oil may have impacted larval survival within a restricted area, the majority of the high quality habitat for each species occurred in areas of the northern GoM unaffected by the oil spill. Therefore, anticipated effects of the DH oil spill on early life survival or reduced larval abundance are expected to be relatively modest, which interestingly is in accord with observed annual patterns of abundance.

Apart from early life processes that directly impact the dispersal and survival of larvae, differences in the demographics (age structure) and abundance (spawning stock biomass) of spawning adults can also influence the abundance of larvae within a given area [31]. Clearly, movement away from or failure to return to a specific spawning area will reduce the production of eggs, and therefore alter patterns of distribution and abundance for larvae within a given region. Detailed information on the demographics of spawning adults of the species examined are not available for the GoM from 2007–2010; however, PAT tagging data provided useful information on the occurrence patterns of one pelagic species (blue marlin) during its spawning period before and after the DH oil spill. Our results showed that the occurrence of adult blue marlin in the area impacted by the DH oil spill was reduced in 2010, possibly indicating that blue marlin did not return to their spawning area in response to altered environmental conditions. Deleterious changes in environmental conditions associated with anthropogenic disturbances (e.g., oil spills, vessel activity, noise) can alter movement patterns causing spatial shifts in the distribution of marine organisms such as sea birds [32]–[33] and free ranging cetaceans [34]–[35]. Direct links between oil and habitat use are equivocal even for marine organisms with advanced cognitive function such as marine mammals [36], and thus behaviorally mediated movement due to surface oil alone is less likely for a marine teleost such as blue marlin. Alternatively, shifts in the occurrence of marine predators, including highly migratory species, are often attributed to changes in prey distributions [37]–[38], and declines in prey availability within the area impacted by the DH oil spill may have been responsible for the increased presence of adult blue marlin in areas outside the impacted area (western and southern GoM) during their presumed spawning period. Regardless of the underlying mechanism, the apparent shift in the spatial occurrence of adult spawners in 2010 likely affected the production and distribution of larvae within our sampling corridor, albeit precaution must be exercised when interpreting these data due to the small sample size available for 2010.

The influence of direct (i.e., larval mortality) and/or indirect (i.e., spatial shifts in spawning stock biomass) effects of the DH oil spill on the abundance and distribution pelagic fishes is currently unresolved, and caution must be exercised when interpreting time-series data that are limited in scope and duration. While larval abundances of all four species examined were lower in 2010 relative to the three-year baseline sample, abundance in 2010 was statistically similar to at least one of the other years surveyed, suggesting that observed declines are within the bounds of expected natural variability. Despite the fact that we cannot explicitly link observed inter-annual trends in the abundance of larvae to the DH oil spill, we can conclude that large areas of high quality habitat of pelagic fishes were exposed to oil contamination. In turn, oil contamination may have contributed to observed temporal patterns of abundance, but the main driver (oil spill vs. natural variability) of observed temporal patterns is unknown.

## Methods

### Early Life Collections

Ichthyoplankton surveys were conducted over a four-year period (2007–2010) from approximately 26.5 to 28.0°N latitude and 87.5 to 92.0°W longitude in the northern GoM. Summer surveys were conducted each year during the same two months (June, July), which corresponds to the primary spawning periods of many pelagic fishes in the GoM [14]. All sampling was conducted during the day (ca. 0700 to 1900 h), and fish larvae were collected with paired neuston nets (2-m width×1-m height frame). At each station, 500 µm and 1200 µm mesh neuston nets were deployed simultaneously. Nets were towed through surface waters for approximately 10 minutes at 2.5 knots, and a small portion of neuston net frame (∼0.25 m) was typically above water. The depth of the surface water sampled during net tows was approximately 0.75 m, and sampling was conducted at approximately 15-km intervals between stations. General Oceanics flowmeters (Model 2030R, Miami, FL) were placed within each neuston net to determine surface area sampled during each tow. Permits for collections of fish larvae were issued by the Highly Migratory Species Division of the National Oceanic and Atmospheric Administration (permits: Billfish-SRP-06-01, Billfish-EFP-07-03, Billfish-EFP-08-03, Billfish-EFP-09-01, Billfish-EFP-10-01).

Fish larvae were sorted in the laboratory, and density and occurrence were determined for blackfin tuna *Thunnus atlanticus* (family Scombridae), blue marlin *Makaira nigricans* and sailfish *Istiophorus platypterus* (family Istiophoridae), and dolphinfish *Coryphaena hippurus* (family Coryphaenidae). Density at each station was determined by combining numbers of individuals in both 500 and 1200 µm mesh nets and adjusted to reflect a sampling depth of 0.75 m. Average volume of water sampled with both nets at each station was approximately 3500 m^3^. Percent occurrence at the species level was also determined and based on the number of stations where selected taxa were present divided by the total number of stations sampled.

Fish larvae were identified visually to family level based on anatomical and morphometric features [39]. Identification to species level was also determined using diagnostic characters; however, identification of smaller larvae (<10 mm standard length, SL) can be problematic for certain species of billfishes, dolphinfishes, and tunas. In response, species-specific multiplex polymerase chain reaction (PCR) assays were used to identify billfish and dolphinfish larvae less than 10 mm SL to the species level. Multiplex PCR assays followed protocols described previously for billfishes [13] and dolphinfishes [40]. Identification of true tuna larvae (genus *Thunnus*) to species was based on sequence variability of mitochondrial DNA [41) and High Resolution Melting (HRM) assays based on fixed nucleotide differences among species [42]. HRM assays were conducted on subsamples taken from each station containing *Thunnus* larvae to determine the abundance of blackfin tuna (*T. atlanticus*) in our collections. Two congeners were also present in our collections (yellowfin tuna *T. albacares* and bluefin tuna *T. thynnus*), and blackfin tuna accounted for nearly 90% of the *Thunnus* larvae collected.

Temporal differences in the densities of larvae collected over the four-year period were compared with a Kruskal-Wallis (rank sums) one-way analysis of variance (ANOVA). This non-parametric method does not assume a normal distribution, which was required due to the large number of zero values in our data set (skewed distribution). Species-specific models were developed for each species in both June and July to investigate temporal differences in larval density for all four species. Non-parametric multiple comparison tests (Wilcoxon method) were run to determine which factor levels or years differed from others. Statistical significance for all tests was based on α = 0.05.

### Habitat Suitability Models

Generalized additive models (GAMs) were used to investigate the influence of environmental conditions on the occurrence of each species. Presence/absence models were fit with a binomial distribution using a logit link function in R software [43]. Occurrence of each species (0 = absent, 1 = present) was modeled against environmental variables over a three-year period (2007–2009). Explanatory variables used in GAMs included oceanographic and biological factors (sea surface temperature, salinity, sea surface height anomaly, chlorophyll *a*, depth), and parameters related to mesoscale oceanographic features (feature classification [cyclonic, anti-cyclonic, open ocean], and distance to closest mesoscale feature) (Table S1). Mesoscale oceanographic features were detected and defined using the Okubo-Weiss algorithm [44], which estimates vorticity and strain from sea surface height images (Aviso) to locate features and then classifies these features as either cyclonic or anticyclonic based on curvature of the ocean surface. Distances from sampling sites to the perimeter of the nearest mesoscale feature were estimated using Spatial Analyst toolbox functions in ArcGIS. Sea surface temperature and salinity were measured at each sampling station using a Sonde 6920 Environmental Monitoring System (YSI Inc.). Other environmental data at sampling stations were extracted from remotely sensed data to match sampling dates and locations [14]. Prior to running GAMs, collinearity among explanatory variables was examined with Spearman rank correlation coefficient (Spearman ρ), and variables used in GAMs were not highly correlated.

A manual backwards stepwise procedure based on minimizing the Akaike Information Criterion (AIC, [45]] was used to select explanatory variables influencing the presence of all four species. Selection of the final model for each species was based on minimizing AIC values, which is a tradeoff between model complexity (number of variables) and fit (based on goodness of fit) [46]. Final GAMs were then used to predict the probability of occurrence for each species based on environmental conditions in 2010. Explanatory variables were linked to prediction grids (cell size = 0.0833°) using the predict.gam function in the mgcv library [47] to estimate the probability of occurrence for each species at each grid point in 2010. For each species-month combination, grid points were converted to a raster surface in ArcGIS 10.0 (ERSI Inc.) and smoothed using bilinear interpolation to visualize suitable habitats. Next, the spatial extent of surface oil from the DH oil spill was viewed in relation to the total available area of high quality habitat (probability of occurrence ≥0.5) to determine the degree of overlap and assess whether this event may have reduced the areal coverage of suitable habitat in the northern GoM in 2010.

### Electronic Tagging of Adults

Pop-up archival transmitting (PAT) tags were deployed opportunistically on blue marlin from sport fishing vessels in the GoM from 2007–2010, following methods previously described [48]. State-space models that implement the Kalman filter algorithm were used to estimate the most probable tracks of blue marlin from light-based location data generated from PAT tags [49]–[50]. These models explicitly account for stochasticity in both measurement (uncertainty in location) and process (fish movement), and further refine movement tracks according to sea surface temperature by comparing tag observations with historical remote sensing data. Reduced state-space models for each blue marlin PAT tag were developed interactively from the light-based locations by removing non-significant parameters as measured by likelihood ratio tests and by considering erroneous locations on land. In cases where the number of observations was small and the pop-up location was relatively close to the deployment location, we used a model with uniform variance structure lacking sea surface temperature parameters and/or advection parameters to estimate a reasonable track solution. Otherwise, the full model often provided the most reasonable solution. Refined estimates from the reduced state-space models were used to approximate daily mean position of blue marlin in the GoM during the presumed spawning period of this species [13].

Daily mean position estimates from the primary spawning season of blue marlin (May–July) were compared before (2007–2009) and after (2010) the DH oil spill. PAT tag data from deployments prior to the DH oil spill (n = 13) ranged from 60 to 365 days. Daily mean positions of adult blue marlin after the spill were based on geo-location estimates generated from 1-year tag deployments on blue marlin (n = 4). These fish were tagged in summer/fall 2009 and the tags released from fish during or after July 2010. Our analysis is based on the assumption that individuals return to the same general spawning area the following year, which is supported by long-term tag deployments on blue marlin and white marlin before or after 2010 (Fig. S5). Gridded probability density was calculated and converted to volume (kernel density based on cell size = 0.01°) using the spatial analyst extension in ArcView 10.0 (ESRI Inc.).

## Supporting Information

Figure S1 **Spatial and temporal variability in the density of pelagic fish larvae collected from 2007 to 2010 for A) blackfin tuna (** ***Thunnus atlanticus*** **), B) blue marlin (** ***Makaira nigricans),*** **C) dolphinfish (** ***Coryphaena hippurus*** **), and D) sailfish (** ***Istiophorus platypterus*** **).** June (red) and July (blue) survey shown and colored lines represent the observed margin of the Loop Current during each sampling trip (coded by color). Density (larvae^.^ 1000 m^−3^) denoted by circle size.
(TIF)

Figure S2 **Response plots from final presence/absence generalized additive models (GAMs) based on June data for A) blackfin tuna (** ***Thunnus atlanticus*** **), B) blue marlin (** ***Makaira nigricans),*** **C) dolphinfish (** ***Coryphaena hippurus*** **), and D) sailfish (** ***Istiophorus platypterus*** **).**
(PDF)

Figure S3 **Response plots from final presence/absence generalized additive models (GAMs) based on July data for A) blackfin tuna (** ***Thunnus atlanticus*** **), B) blue marlin (** ***Makaira nigricans),*** **C) dolphinfish (** ***Coryphaena hippurus*** **), and D) sailfish (** ***Istiophorus platypterus*** **).**
(PDF)

Figure S4 **Map showing the region closed to fishing on June 21, 2010 by the National Oceanic and Atmospheric Administration due to the Deepwater Horizon oil spill.**
(TIF)

Figure S5 **Long-term tracks (ca. 1 year) of individual A) white marlin (** ***Kajikia albida*** **) and B) blue marlin (** ***Makaira nigricans)*** **from pop-up archival transmitting (PAT) tags deployed in the Gulf of Mexico.** Blue shading depicts kernel density (95% volume of utilization distribution shown) based on daily mean positions the first month of the deployment (dark blue) and the same month the next year (light blue). Colored symbols represent quarterly geo-location estimates for each individual over the deployment period and all geo-location data were generated using state-space models that implemented the Kalman filter algorithm from light-based location data generated from PAT tags. Estimated weights of white marlin (ID: WM-09-01) and blue marlin (ID BM-10-02) shown here were 30 and 120 kg, respectively.
(TIF)

Table S1 (DOCX)
