# Supplementary figures and images for: Spatial, Temporal, and Habitat-Related Variation in Abundance of Pelagic Fishes in the Gulf of Mexico: Potential Implications of the Deepwater Horizon Oil Spill
Source: PLoS One. 2013 Oct 10;8(10):e76080. doi: 10.1371/journal.pone.0076080 (PMC3794940; doi:10.1371/journal.pone.0076080)

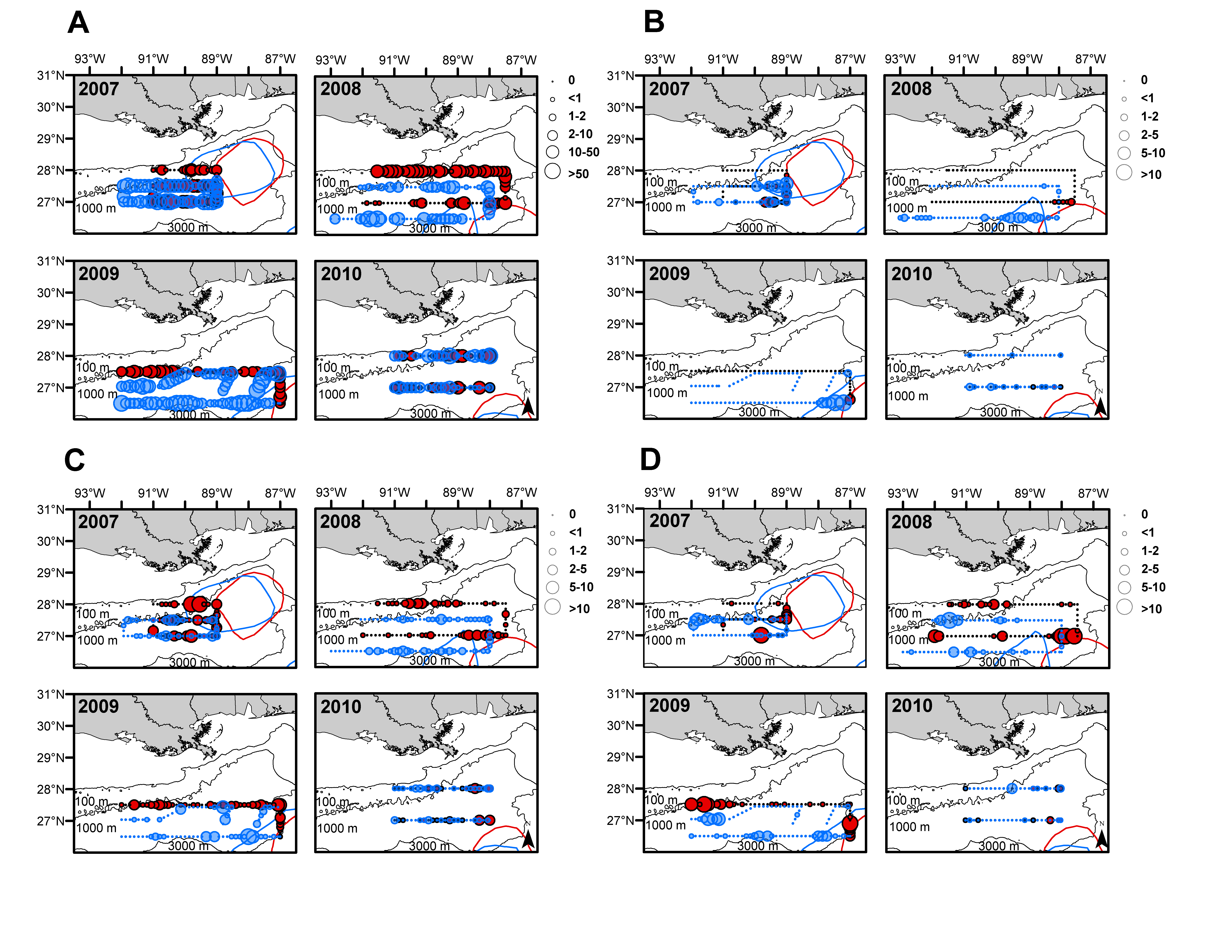

Supplement: Figure S1 — Spatial and temporal variability in the density of pelagic fish larvae collected from 2007 to 2010 for A) blackfin tuna ( Thunnus atlanticus ), B) blue marlin ( Makaira nigricans), C) dolphinfish ( Coryphaena hippurus ), and D) sailfish ( Istiophorus platypterus ). June (red) and July (blue) survey shown and colored lines represent the observed margin of the Loop Current during each sampling trip (coded by color). Density (larvae. 1000 m−3) denoted by circle size. (TIF) [file pone.0076080.s001.tif]

**A**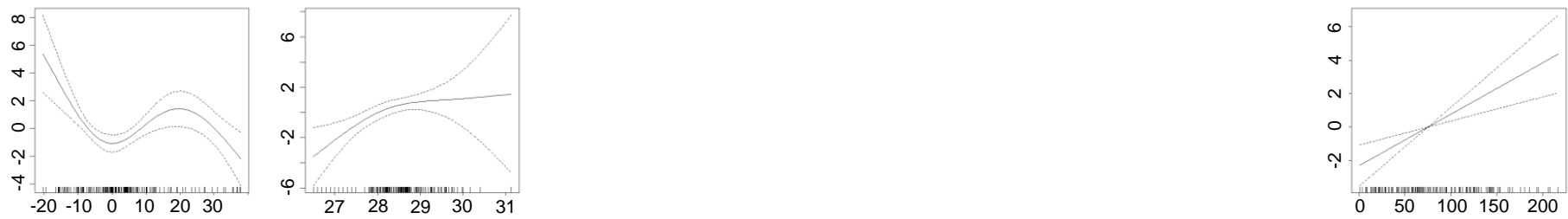**B**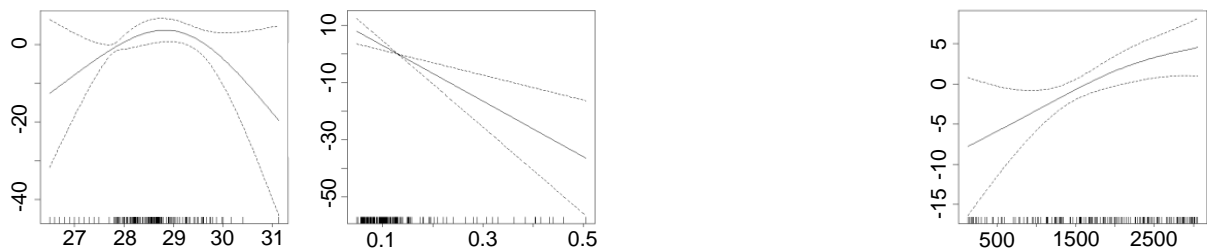**C**

Additive effect

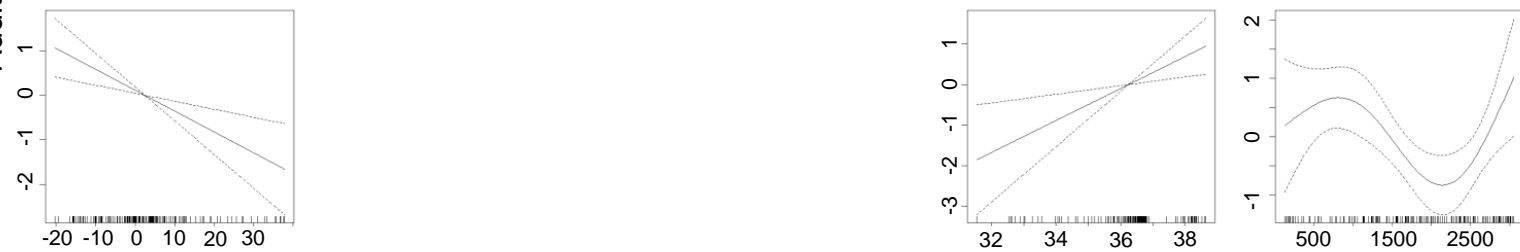**D**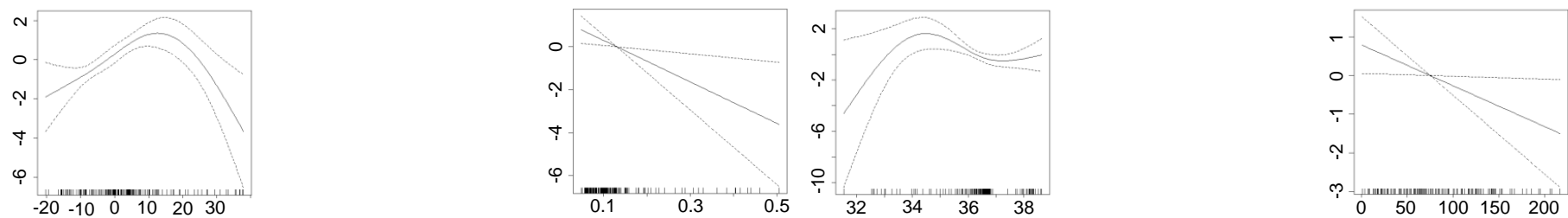

SSHA (cm)

SST (°C)

Chl a (mg m<sup>-3</sup>)

Salinity

Depth (m)

Distance to  
feature (km)

Supplement: Figure S2 — Response plots from final presence/absence generalized additive models (GAMs) based on June data for A) blackfin tuna ( Thunnus atlanticus ), B) blue marlin ( Makaira nigricans), C) dolphinfish ( Coryphaena hippurus ), and D) sailfish ( Istiophorus platypterus ). (PDF) [file pone.0076080.s002.pdf]

**A**

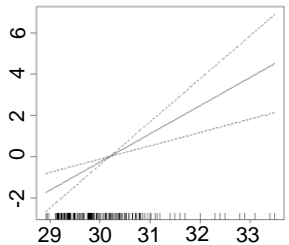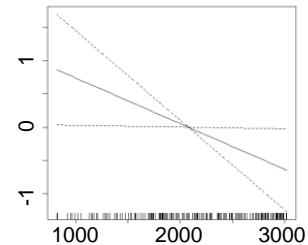

**B**

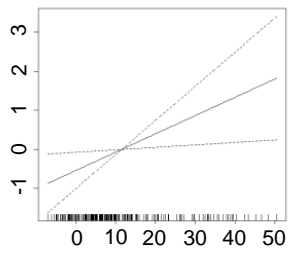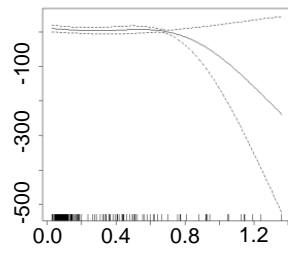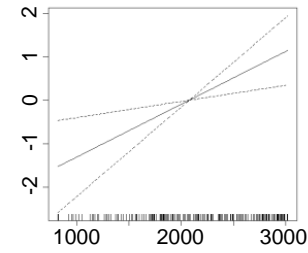

**C**

Additive effect

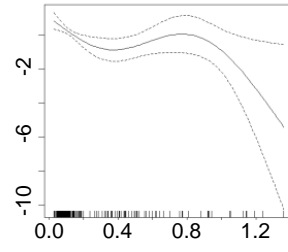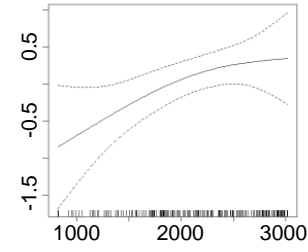

**D**

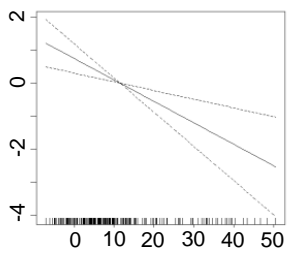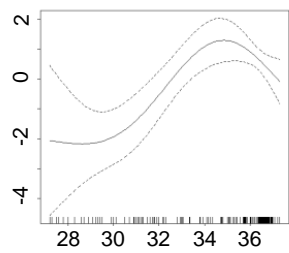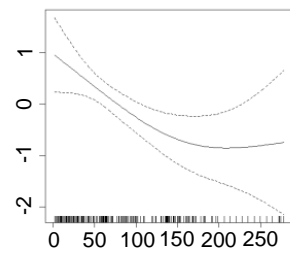

**SSHA (cm)**

**SST (°C)**

**Chl a (mg m<sup>-3</sup>)**

**Salinity**

**Depth (m)**

**Distance to  
feature (km)**

Supplement: Figure S3 — Response plots from final presence/absence generalized additive models (GAMs) based on July data for A) blackfin tuna ( Thunnus atlanticus ), B) blue marlin ( Makaira nigricans), C) dolphinfish ( Coryphaena hippurus ), and D) sailfish ( Istiophorus platypterus ). (PDF) [file pone.0076080.s003.pdf]

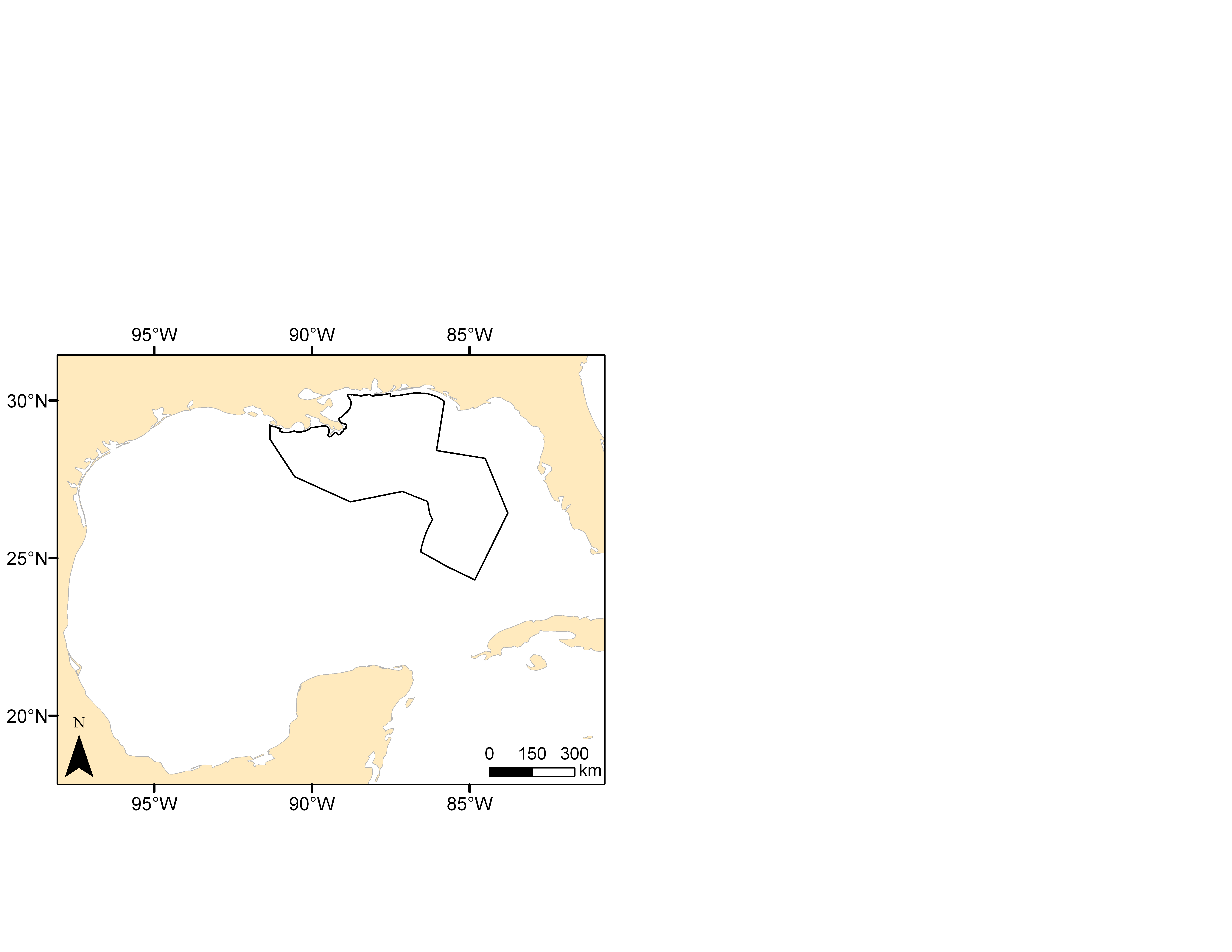

Supplement: Figure S4 — Map showing the region closed to fishing on June 21, 2010 by the National Oceanic and Atmospheric Administration due to the Deepwater Horizon oil spill. (TIF) [file pone.0076080.s004.tif]
