# Supplementary material for: Spatial, Temporal, and Habitat-Related Variation in Abundance of Pelagic Fishes in the Gulf of Mexico: Potential Implications of the Deepwater Horizon Oil Spill
Source: PLoS One. 2013 Oct 10;8(10):e76080. doi: 10.1371/journal.pone.0076080 (PMC3794940; doi:10.1371/journal.pone.0076080)
Supplement: Table S1 — (DOCX) [file pone.0076080.s006.docx]

**Table S1**: Summary information on remotely sensed datasets used for explanatory variables in GAMs. Descriptions and sources of each included. Data for all variables except depth were obtained using the Marine Geospatial Ecology Toolbox (version 0.8a42) in ArcGIS. Note: sea surface temperature and salinity measured at each station during all surveys.
